# Juvenile Reformatories

**Published:** 1898-05-28

**Authors:** 


### Modern Sociology.

### JUVENILE REFORMATORIES.

The best method of dealing with those sections of our population who come under the ominous designation of the criminal classes is at present occupying the anxious attention of the Government, as may be known by the progress of the Prisons Bill and other measures of reform already enforced. As yet, however, beyond the passing of the "First Offenders" Act, no radical improvement has been effected as regards the treatment of criminal children. Yet it is beyond measure the most important of all the branches of this vast subject—the very root and spring of the crime which in so many different forms devastates our country. Victor Hugo has pointed to the truth in this matter in one concise and most telling sentence: "Tous les crimes de l'homme commencent au vagabondage de l'enfant." And it is so; to check the first seeds of vice in those still young and pliable enough to be amenable to good influences is the only effort for the amelioration of the lower stratum of society on which we may count with any hope of success. Now, it must be owned that this is one of the matters which they manage better in France than we have as yet been able here; and this truth is forcibly shown in an admirable and exhaustive article on "Juvenile Reformatories in France" which has lately been contributed by Mr. E. R. Spearman to the *Fortnightly Review*. We should be glad indeed if we could follow him through all the interesting details he gives of the many-sided organisation for the reformation of young offenders which has been established by the "Comité de Defense des Enfants Traduits en Justice"; but, apart from the restrictions of space, our object in endeavouring to influence public opinion through these columns is in order that one especial measure now enforced in France, may be brought prominently before the notice of the authorities at the Home Office. We allude to the system of placing male criminal children, as well as females, under the care of women only. We have our reformatories and industrial schools in this country, and we are most ready to admit the anxiety especially manifested of late by the Government, to do the best that can possibly be done in the department of juvenile crime. But those most competent to judge, such as the governors of prisons and others who have to deal with law breakers old and young, are clearly of opinion that the reformatories for boys managed exclusively by men are not a success—at least, as regards their after results. The discipline may be well maintained, and every effort is undoubtedly made by the conscientious masters to counteract the inevitable evils of the association of very young boys with those more advanced in crime; but when the time comes for the youths to leave these enclosures and go out into the world, it is too often found that their characters have not been moulded, nor their wills trained in a right direction, as might have been expected from the good influences brought to bear upon them. We firmly believe that the remedy for this disappointing result would be found in the appointment of women, instead of men, to take the entire charge of the reformatories and schools for young criminal boys.

This opinion is emphatically supported by the practical results of the system as it has been carried out in France since the year 1876. Mr. Spearman not only enlarges strongly on the advantages of this plan, but gives the figures which prove its efficacy beyond question—as may be seen by the passages we now quote from his admirable article: "It is a well-known fact that women exercise over boys, even the very worst, if they are handed to their care young enough, an influence the secret of which the best of men never possess. The French reformatory system fully recognises this, and all children under 12 years of age are sent to 'reform schools,' where they will be subject to this benign female influence. There are three such 'reform schools' in France. The Government establishment consists of three farms, about a mile apart. To the first of these, which is under the entire care of ladies, the children are sent on arrival; at thirteen years of age they are passed to the second farm, and at sixteen to the third; each group is thus kept entirely separated according to age. These establishments are managed by *religieuses*. The only men on the premises are the farm bailiff and a man to look after the stables. The whole work of the farm is directed by the Sisters. They receive boys into their school as young as six, but they decline to receive any after they have attained their twelfth year. These youths, of whom there are more than 400, remain under the sole charge of the Sisters until their departure at nearly 20 years of age. The happy influence of these ladies on young children remains after they have become men—have been soldiers and have married and settled in the neighbourhood. The choice of the staff is of the utmost importance, for the gentle, but firm, authority of well-chosen women is never contested; it is accepted with affection and respect."

We cannot too strongly advocate the establishment of this system in England, and we earnestly hope that the adoption of the plan may speedily engage the attention of the authorities at the Home Office. There are, however, one or two modifications of the French system which might be advisable in this country. Mr. Spearman rightly mentions the great importance of a wise choice for the staff of workers—in France they are exclusively *religieuses*, and sisters belonging to the Church of England would, no doubt, readily be found to undertake similar posts, but we do not think it would be desirable that their numbers should be drawn upon for the purpose. The higher education of women has supplied us with a vast assemblage of clever, capable persons, who can efficiently take the management of high schools and hospitals and other important institutions, and by whom our new "reform schools" could be admirably conducted; we do not mean in the least to disparage English sisterhoods, which accomplish much excellent work, but they are very rigid and most uncompromising as to the enforcement of their own numerous rules, and their quiet, demure inmates would certainly not be so attractive to boys as the cheerful, animated French nuns.

Another point on which it would be well to deviate from the French rule, would be an increase in the age at which boys would be admitted to reform schools. To take them only under the age of twelve years would exclude many little victims to evil surroundings who would probably yield readily enough to the influence of good and wise women over them. These, however, are only minor considerations, while it is eminently desirable that the system, as a whole, should be adopted in this country as soon as possible.

